# The feasibility of a randomised controlled trial to compare the cost-effectiveness of palliative cardiology or usual care in people with advanced heart failure: Two exploratory prospective cohorts

**DOI:** 10.1177/0269216318763225

**Published:** 2018-04-24

**Authors:** Miriam J Johnson, Paula McSkimming, Alex McConnachie, Claudia Geue, Yvonne Millerick, Andrew Briggs, Karen Hogg

**Affiliations:** 1 Wolfson Palliative Care Research Centre, Hull York Medical School and University of Hull, Hull, UK; 2 Robertson Centre for Biostatistics, Institute of Health & Wellbeing, University of Glasgow, Glasgow, UK; 3 Health Economics and Health Technology Assessment, Institute of Health & Wellbeing, University of Glasgow, Glasgow, UK; 4 Glasgow Caledonian University, British Heart Foundation, NHS Greater Glasgow and Clyde, Glasgow, UK; 5 William R Lindsay Chair of Health Economics (Health Economics and Health Technology Assessment), Institute of Health & Wellbeing, University of Glasgow, Glasgow, UK; 6 Glasgow University, Glasgow, UK

**Keywords:** Palliative care, effectiveness, heart failure, feasibility studies

## Abstract

**Background:**

The effectiveness of cardiology-led palliative care is unknown; we have insufficient information to conduct a full trial.

**Aim:**

To assess the feasibility (recruitment/retention, data quality, variability/sample size estimation, safety) of a clinical trial of palliative cardiology effectiveness.

**Design:**

Non-randomised feasibility.

**Setting/participants:**

Unmatched symptomatic heart failure patients on optimal cardiac treatment from (1) cardiology-led palliative service (caring together group) and (2) heart failure liaison service (usual care group).

**Outcomes/safety:**

Symptoms (Edmonton Symptom Assessment Scale), Kansas City Cardiomyopathy Questionnaire, performance, understanding of disease, anticipatory care planning, cost-effectiveness, survival and carer burden.

**Results:**

A total of 77 participants (caring together group = 43; usual care group = 34) were enrolled (53% men; mean age 77 years (33–100)). The caring together group scored worse in Edmonton Symptom Assessment Scale (43.5 vs 35.2) and Kansas City Cardiomyopathy Questionnaire (35.4 vs 39.9). The caring together group had a lower consent/screen ratio (1:1.7 vs 1: 2.8) and few died before approach (0.08% vs 16%) or declined invitation (17% vs 37%).

**Data quality:**

At 4 months, 74% in the caring together group and 71% in the usual care group provided data. Most attrition was due to death or deterioration. Data quality in self-report measures was otherwise good.

**Safety:**

There was no difference in survival. Symptoms and quality of life improved in both groups. A future trial requires 141 (202 allowing 30% attrition) to detect a minimal clinical difference (1 point) in Edmonton Symptom Assessment Scale score for breathlessness (80% power). More participants (176; 252 allowing 30% attrition) are needed to detect a 10.5 change in Kansas City Cardiomyopathy Questionnaire score (80% power; minimum clinical difference = 5).

**Conclusion:**

A trial to test the clinical effectiveness (improvement in breathlessness) of cardiology-led palliative care is feasible.

**What is already known about the topic?**

- Compared with people with cancer, those with advanced heart failure have equivalent palliative care needs with poorer access to specialist palliative care.
- A palliative care approach led by cardiology teams and access to specialist palliative care according to need rather than prognosis is recommended but poorly implemented.
- The evidence base for palliative care in heart failure is less robust than that for cancer and there are no published prospectively collected data for cardiology-led palliative care services.

**What this paper adds?**

- Novel prospectively collected data for cardiology-led palliative care services which show that a future trial of cardiology-led palliative care is feasible in terms of recruitment to a clinical study (note: willingness to randomisation was not tested), data quality and sample size.
- Data generating a hypothesis that cardiology-led palliative care may safely improve advance care planning and patients’ understanding of their condition, enable care at home and reduce costs.

**Implications for practice, theory or policy**

- Findings should be tested in an adequately powered randomised controlled trial.
- Future studies should identify and randomise patients with more severe or complex palliative care needs to ensure optimal use of finite specialist palliative care resources.

## Introduction

Compared with people with cancer, those with advanced heart failure have equivalent palliative care needs with poorer access to specialist palliative care.^1–6^ Palliative care is recommended,^7–10^ but poorly implemented.^11–15^

Phase III trials of specialist palliative care for people with heart failure^16–19^ report cost-effective^20,21^ benefit with specialist palliative care without compromising survival. However, most palliative concerns in people with heart failure can be managed by the usual care team using a patient-centred approach with access to specialist palliative care for complex issues only.^22,23^ Previous integrated service descriptions do not include patient-reported outcomes,^24–27^ and the effectiveness of cardiology-led palliative care is untested.

A partnership between two charities (Marie Curie and British Heart Foundation) and a Scottish NHS health board developed cardiology-led models of heart failure palliative care (‘The Caring Together (CT) Programme’^28^).

The study objectives were to assess the feasibility of a future randomised controlled trial (RCT) in terms of (1) recruitment/retention, (2) data quality, (3) variability/sample size estimation and (4) safety.

## Methods

Expanded methods are available online.

### Study design

This prospective feasibility cohort single-site study (data collection at baseline, 2 and 4 months) used two heart failure outpatient groups. The participants continued 2 monthly measures until the last recruit completed 4 months’ follow-up.

Ethical (NRES Committee London – Camberwell St Giles. REF 14/LO/1813; 07.10.2014) and institutional approvals were obtained prior to recruitment. The participants gave written informed consent.

### Participants

Participants were adults with symptomatic heart failure (reduced or normal left ventricular ejection fraction; HFrEF, HFnEF) and family carers. Participants with at least one CT clinic attendance formed the caring together group (CTG). A convenience unmatched usual care group (UCG) was formed from heart failure liaison nurse service patients fulfilling CT criteria but who had not been referred. The risk of contamination was deemed to be minimal.^29^

### Caring together intervention

Box 1 shows the referral criteria and components. Collaborative primary and secondary care is led by a consultant cardiologist and a heart failure palliative care nurse consultant, through a palliative cardiology multi-disciplinary team (MDT).

**Box 1.** Caring together components (adapted, Caring Together Manualisation Report, 2014).^28^

I. Patient identification and referral • Diagnosis of heart failure^a^ (NYHA III or IV). • Distressing or debilitating symptoms despite optimal medical therapy. Supportive or palliative care needs (physical, social, emotional, spiritual or psychological). II. Holistic assessment • Cardiology review. • Holistic assessment: review with patient and carer needs, identification of individually tailored solutions. III. Care management and coordination • Care manager assigned (usually heart failure nurse specialist) as the main contact for care management, information, advice and support. IV. Training and education • Training provided to stakeholders delivering services within the programme. • Shared learning between specialties (palliative care/cardiology and service delivery settings, community/acute care). V. Multi-disciplinary work and joint working • Joint working and care coordination across teams (community, out-of-hours care, acute care): problems identified during the holistic assessment may trigger referral to other agencies or other MDT members, including primary care. • Care manager coordinates care with the MDT and actions additional referrals if required. • Individually tailored medical anticipatory care plan (MACP), including tailored anticipated medical interventions, such as cardiopulmonary resuscitation status decisions, device management and place of care/death shared with all members of the healthcare team including out-of-hours teams and specialist palliative care as needed.

a Patients with reduced or normal left ventricular function are eligible.

### Usual care

The liaison nurse service and other consultant cardiologists’ clinics provide education, self-care promotion, medical therapy up-titration and patient/family support (HFrEF only).

### Study measures

1. Recruitment: screen-to-consent ratio, reasons for non-consent, recruitment rate, retention.
2. Data quality of protocol measures.
  (a) Demographic and clinical measures at baseline: New York Heart Association (NYHA) status;^30^ Australian-modified Karnofsky Performance Status (AKPS);^31^ Charlson et al.’s^32^ comorbidity index; medication-optimised cardiac treatment; pulse, heart rate, presence/absence of oedema and jugular venous pressure; urea and electrolytes; and echocardiogram (HFrEF and HFnEF; for HFrEF, ‘mild, moderate, severe’, ejection fraction).
  (b) Patient measures at baseline and follow-up: AKPS, Edmonton Symptom Assessment Scale – revised (ESAS-r),^33^ Kansas City Cardiomyopathy Questionnaire (KCCQ-12),^34–36^ EuroQol EQ-5D-5L,^37^ Hospital Anxiety and Depression Scale (HADS),^38^ patient understanding (Likert), health service utilisation and costs (including medication) and Zarit 6 caregiver burden (carers).^39^
  (c) Clinical measures at baseline and follow-up: survival, clinical documentation of anticipatory care planning (ACP).
3. Candidate primary outcome data variability for power estimation.
4. Safety: survival (6 months) and outcomes (4 months).

Data were not collected on willingness to be randomised.

### Statistical analysis

Data are presented using descriptive statistics. Sample size estimations were conducted based on variability data around candidate primary outcomes.

Safety assessment: (1) Cox proportional hazards models were used for between-group survival differences and (2) Group 4 month outcomes comparison by study entry group used two-sample *t* tests (continuous) and Fisher’s exact tests (categorical), unless otherwise stated. No adjustments were made for multiple comparisons. Missing data were not imputed. A ‘per-protocol’ (PP) repeated-measures model analysis excluded UCG participants referred to CT. Two random-effects repeated-measures regression models with (1) the KCCQ-12 and (2) the EQ-5D-5L, as the dependent outcomes adjusted for age, sex and baseline ESAS-r scores, were used. The estimated between-group differences were reported with 95% confidence intervals (CIs) and *p* values. PP analysis was completed for the initial repeated-measures model (data available).

Incremental costs and outcomes associated with CTG were determined. Resource use items included health service utilisation and medication. Analyses were conducted using SAS for Windows version 9.3. Formal power calculation was inappropriate.

## Results

A total of 77 participants (43 CTG and 34 UCG) were recruited (8 April to 18 December 2015). A follow-up of 4 months is presented except for the repeated-measures model, survival and health service utilisation (8 months) and study flow (10 months).

### Feasibility measures

Table 1 shows the patient flow. One UCG participant consented for every 2.8 screened versus 1 per 1.4 CTG screened. Fewer in the CTG died before approach (0.08% vs 16%) or declined invitation (17% vs 37%).

**Table 1.** Patient flow.

	CTG, *n* (%)	UCG, *n* (%)
Screening	62	94
Declined	11 (17)	35 (37)
Died before approach	5 (0.08)	15 (16)
Unable to consent	0	4
Admitted to hospital	1	3
Other	2	2
Baseline	43/62 (69)	34/94 (36)
Attrition following baseline		
Died	4	2
Withdrew consent	1	0
Deterioration	1	2
Failed to attend	1	0
2-month follow-up	36/43 (84)	30/34 (88)
Attrition following 2 months		
Died	2	1
Withdrew consent	0	2
Deterioration	2	0
Failed to attend	0	1
4-month follow-up	32/43 (74)	24/34 (71)
Longer term follow-up for those recruited earlier in the study		
6-month follow-up	19	8
8-month follow-up	11	5
Patient status at 10-month follow-up		
Attended	1	0
Died	9	5
Withdrew consent	3	4
Not possible^a^	29	25
Lost to follow up	1	0

CTG: caring together group; UCG: usual care group.

* Patients recruited later in the study would not have sufficient follow-up time to complete visits beyond 4 months after the recruitment date.

Most attrition was due to death or deterioration; those providing follow-up data had better performance status, symptom scores and quality of life (QoL) at baseline (data available). Data quality was otherwise good (Tables 2 and 3).

**Table 2.** Outcomes at 4 months.

	Values at 4 months			Change from baseline at 4 months		
	CTG, *N* = 32	UCG, *N* = 24	*P* value	CTG, *N* = 32	UCG, *N* = 24	*P* value
Health status and QoL						
MD, *n*	0	0	–	0	0	–
AKPS, median (IQR)	65 (60, 70)	60 (60, 70)	0.790**	0 (0, 0)	0 (–10, 0)	0.687**
MD, *n*	2	1	–	2	1	–
EQ-5D, mean (SD)	0.586 (0.227)	0.663 (0.250)	0.255	0.1 (0.2)	0.1 (0.3)	0.385*
MD, *n*	2	0	–	2	0	–
EQ-5D VAS, mean (SD)	56.2 (22.0)	61.7 (23.6)	0.386	3.5 (28.6)	–4.0 (22.6)	0.901*
KCCQ-12 score, mean (SD)	42.25 (21.71)	52.92 (24.40)	0.068	5.7 (20.3)	12.4 (25.2)	0.064*
Symptoms						
ESAS-r score, mean (SD)	39.23 (16.84)	28.09 (17.51)	0.022	−1.5 (14.8)	−5.6 (16.6)	0.046*
MD, *n*	3	0	–	3	0	–
HADS–anxiety, mean (SD)	5.7 (4.3)	4.0 (5.4)	0.232	−2.0 (3.6)	−3.3 (3.7)	0.103*
HADS–depression, mean (SD)	6.8 (4.3)	5.8 (4.3)	0.408	−0.5 (3.8)	−0.5 (4.1)	0.679*
Caregivers						
Caregivers, *n* (MD)	14 (1)	2 (3)	–	14 (1)	2 (3)	–
Zarit caregiver, mean (SD)	10.7 (6.4)	3.5 (2.1)	0.027	0.1 (3.1)	–4.0 (2.8)	0.106*

CTG: caring together group; UCG: usual care group; QoL: quality of life; MD: missing data; SD: standard deviation; N: number; IQR: inter-quartile range; AKPS: Australian Karnofsky Performance Scale; EQ-5D: EuroQol-5 dimension scale; ESAS-r: Edmonton Symptom Assessment Scale – revised; KCCQ-12: Kansas City Cardiomyopathy Questionnaire; HADS: Hospital Anxiety and Depression Scale.

* Adjusted for baseline; **Wilcoxon–Mann–Whitney test.

**Table 3.** Outcomes at 4 months regarding understanding and anticipatory care planning.

	CTG, *N* = 32	UCG, *N* = 24	*P* value
Understanding and anticipatory care planning			
Missing data (excluding drop-outs)	2	0	–
Patients’ understanding of care, *n* (%)			<0.001*
Not at all	0 (0.0)	0 (0.0)	
A little	4 (13.3)	11 (45.8)	
Fairly well	9 (30.0)	10 (41.7)	
Very well	15 (50.0)	3 (12.5)	
Completely	2 (6.7)	0 (0.0)	
Missing data (excluding drop-outs), *n*	0	0	–
DNAR-CPR: Evidence of discussion with patient in case notes, *n* (%)	28 (77.8)	7 (24.1)	<0.001
DNAR-CPR: Evidence of discussion with carer in case notes, *n* (%)	21 (58.3)	4 (13.8)	<0.001
CARE: Evidence of discussion with carer in case notes, *n* (%)	29 (80.6)	9 (31.0)	<0.001
CARE: Evidence of discussion with patient in case, *n* (%)	23 (63.9)	4 (14.3)	<0.001
Documented preferred place of care^a^, *n* (%)			0.184
Home	24 (82.8)	6 (66.7)	
Care home	0 (0.0)	1 (11.1)	
Hospital	5 (17.2)	2 (22.2)	
Hospice	0 (0.0)	0 (0.0)	

CTG: caring together group; UCG: usual care group; MD: missing data; *N*: number; DNAR-CPR: do not attempt cardiopulmonary resuscitation.

a Number (%) of those who provided a response to this question (CTG: *n* = 29; UCG: *n* = 9).

* Wilcoxon–Mann–Whitney test.

## Baseline characteristics

Both groups were on optimal tolerated cardiac treatment.

There were clinically important between-group differences (Tables 4 and 5). Nearly all UCG participants had HFrEF, but half of the CTG participants had HFnEF (Table 4). CTG patients had worse NYHA class, symptoms (ESAS-r) and quality of life (KCCQ-12), and less deprivation. Most CTG participants had ACP documented and better understanding.

**Table 4.** Baseline demographic characteristics.

	All patients, *N* = 77	CTG, *N* = 43	UCG, *N* = 34	*P* value
MD	0	0	0	–
Age in years, mean (SD) Range	77.0 (11.9) 33–100	75.8 (12.3) 33–100	78.4 (11.3) 54–96	0.339
Gender: male, *n* (%)	41 (53.2)	24 (55.8)	17 (50.0)	0.651
SIMD quintiles; MD, *n*	2	2	0	
Most deprived, *n* (%)	27 (36.0)	12 (29.3)	15 (44.1)	0.564
2	9 (12.0)	7 (17.1)	2 (5.9)	
3	9 (12.0)	5 (12.2)	4 (11.8)	
4	8 (10.7)	6 (14.6)	2 (5.9)	
Least deprived	4 (5.3)	2 (4.9)	2 (5.9)	
Hospital admissions				
Admitted due to HF within the past 6 months, *n* (%)	30 (39.0)	14 (32.6)	16 (47.1)	0.242
Admitted to hospital in the past 1 month, *n* (%)	11 (14.3)	4 (9.3)	7 (20.6)	0.199
Number of nights in hospital in the past 1 month, *n* (SD)	6.3 (22.1)	4.5 (21.0)	8.6 (23.6)	0.429
NYHA status, *n* (%)				
Class I	0 (0.0)	0 (0.0)	0 (0.0)	0.067*
Class II	3 (3.9)	0 (0.0)	3 (8.8)	
Class III	70 (90.9)	40 (93.0)	30 (88.2)	
Class IV	4 (5.2)	3 (7.0)	1 (2.9)	
Echocardiography				
Echo available, *n* (%)	62 (80.5);	32 (74.4)	30 (88.2)	0.156
Echo LVSD, *n* (%)	45 (72.6)	16 (50.0)	29 (96.7)	<0.001
Ejection fraction; MD, *n*	21	15	6	–
Mean, % (SD)	42.1 (16.4)	46.5 (19.4)	37.6 (11.3)	0.041
Urea and electrolytes				
Sodium; MD, *n*	1	1	0	–
Mean, mmol/l (SD) Range	139 (3.3) 130–148	138 (3.9) 130–148	139 (2.5) 134–144	0.323
Potassium; MD, *n*	2	2	0	
Mean, mmol/l (SD) Range	4.2 (0.4) 3.0–5.1	4.2 (0.4) 3.0–5.1	4.3 (0.5) 3.2–5.1	0.869
eGFR; MD, *n*	1	1	0	
Mean, mL/min (SD) Range	46.4 (14.4) 10–77	46.7 (15.3) 10–60	46.0 (13.5) 20–77	0.834
Charlson comorbidity index; median (IQR) Range	7 (6, 8) 2–14	7 (6, 8) 2–14	8 (6, 9) 4–12	0.029*
Cardiac medication, *n* (%)				
ACE inhibitor	28 (36.4)	12 (27.9)	16 (47.1)	0.099
ARB	11 (14.3)	6 (14.0)	5 (14.7)	1.000
Beta-blocker	47 (61.0)	21 (48.8)	26 (76.5)	0.019
Aldosterone blocker	25 (35.7)	18 (48.6)	7 (21.2)	0.024
Aldosterone blocker: spironolactone	21 (27.3)	15 (34.9)	6 (17.6)	0.124
Aldosterone blocker: eplerenone	4 (5.2)	3 (7.0)	1 (2.9)	0.626

CTG: caring together group; UCG: usual care group; MD: missing data; SD: standard deviation; *N*: number; SIMD: standardised index of multiple deprivation; HF: heart failure; IQR: inter-quartile range; NYHA: New York Heart Association; LVSD: left ventricular systolic dysfunction; eGFR: estimated glomerular filtration rate; ACE: angiotensin-converting enzyme; ARB: angiotensin II receptor blocker.

* Wilcoxon–Mann–Whitney test.

**Table 5.** Baseline outcome measures.

No missing data	All, *N* = 77	CTG, *N* = 43	UCG, *N* = 34	*P* value
Health status and quality of life				
EQ-5D score, mean (SD) Range	0.524 (0.288) –0.170 to 0.951	0.517 (0.318) –0.170 to 0.951	0.532 (0.250) –0.027 to 0.951	0.828
EQ-5D VAS, mean (SD) Range	55.2 (26.0) 5 to 100	50.5 (26.8) 5 to 99	61.2 (24.0) 10 to 100	0.070
KCCQ-12 score, mean (SD) Range	37.36 (26.47) 0 to 100	35.37 (25.57) 0 to 88	39.89 (27.75) 0 to 100	0.465
Symptoms				
ESAS-r score, mean (SD) Range	39.81 (21.14) 0 to 82	43.45 (21.56) 4 to 82	35.21 (19.96) 0 to 78	0.087
HADS–anxiety, mean (SD) Range	7.7 (5.3) 0 to 20	7.8 (4.9) 0 to 19	7.5 (5.9) 0 to 20	0.786
HADS–depression, mean (SD) Range	7.8 (4.5) 0 to 17	8.5 (4.6) 0 to 17	6.9 (4.4) 0 to 16	0.140
Caregivers				
Zarit caregiver, mean (SD) Range	11.0 (5.3) 0 to 20	11.2 (5.3) 0 to 19	10.6 (5.8) 2 to 20	0.820
Understanding and advance care planning				
Patients’ understanding of care, *n* (%)				
Not at all	5 (6.5)	1 (2.3)	4 (11.8)	0.003*
A little	32 (41.6)	14 (32.6)	18 (52.9)	
Fairly well	24 (31.2)	15 (34.9)	9 (26.5)	
Very well	15 (19.5)	12 (27.9)	3 (8.8)	
Completely	1 (1.3)	1 (2.3)	0 (0.0)	
DNAR-CPR: Evidence of discussion with patient in case notes, *n* (%)	38 (49.4)	33 (76.7)	5 (14.7)	<0.001
DNAR-CPR: Evidence of discussion with carer in case notes, *n* (%)	24 (31.2)	22 (51.2)	2 (5.9	0.001
Documented ACP discussion (patient), *n* (%)	35 (45.5)	29 (67.4)	6 (17.6)	0.001
Documented ACP discussion (carer), *n* (%)	26 (33.8)	23 (53.5)	3 (8.8)	<0.001
Documented preferred place of care, *n* (%)				
Home	30 (85.7)	25 (86.2)	5 (83.3)	0.634*
Care home	0 (0.0)	0 (0.0)	0 (0.0)	
Hospital	4 (11.4)	3 (10.3)	1 (16.7)	
Hospice	1 (2.9)	1 (3.4)	0 (0.0)	

CTG: caring together group; UCG: usual care group; SD: standard deviation; *N*: number; EQ-5D: EuroQol-5 dimension scale; ESAS-r: Edmonton Symptom Assessment Scale – revised; KCCQ-12: Kansas City Cardiomyopathy Questionnaire; HADS: Hospital Anxiety and Depression Scale; DNAR-CPR: do not attempt cardiopulmonary resuscitation; ACP: anticipatory care plan.

* Wilcoxon–Mann–Whitney test.

More UCG participants had prior admissions (47.1% vs 32.6%). All CTG participants had attended CT at least once (median: 8 months; inter-quartile range (IQR): 2–21 months).

### Group comparison at follow-up

Overall, the ESAS-r and KCCQ-12 scores improved. At 4 months, adjusted symptom improvement was greater in the UCG (0.046). ACP documentation and understanding remained better in the CTG (*p* < 0.001). Two UCG participants were transferred to CTG, but there were no differences in the PP analysis findings.

CTG participants had fewer nights in hospital, but more GP visits (eTable 1). CT participants commented on excellent, individually tailored, coordinated care. UCG participants commented on fragmented care, poor communication and different unfamiliar doctors.

There was no between-group difference in survival (eFigure 1 online; time to death).

### Repeated-measures model

There were no between-group differences in EQ-5D-5L (*p* = 0.50) or KCCQ-12 (*p* = 0.08) at 4 months after adjustment for age, sex or baseline individual ESAS-r (eFigure 2 online).

### Health service use and costs

Differences in health service use are shown in eTable 1. Estimated differences in costs show that the average healthcare costs reduced by £785 in CTG. This is statistically insignificant and subject to considerable uncertainty.

### Sample size calculation for the main trial

Important clinical differences for KCCQ-12^40^ and ESAS (breathlessness)^41^ were used. UCG was taken as the population of interest and ESAS (breathlessness) as the symptom most highly correlated with KCCQ-12 (data available). eTable 2 shows the estimated sample sizes (80% and 90% power; alpha 0.05). To detect a difference of 10.5 points (KCCQ-12) and 1 point (ESAS breathlessness), the sample sizes of 176 and 141, respectively, are needed (30% attrition; 252 and 202).

## Discussion

### Feasibility outcomes

It is feasible to recruit and collect data for a clinical study. As reported previously in palliative studies,^42^ attrition was high (27% at 4 months) mostly due to death or deterioration. Further feasibility work should test the willingness to be randomised.

### Strengths and limitations

This the first study to provide patient report and cost-effectiveness data from a cardiology-led palliative heart failure service. Other studies report the components only (ACP, patient-centred decisions)^43^ or historical controls.^27^

The major limitations are as follows: (1) non-randomisation, (2) cost neutrality was assumed and opportunity costs were not considered, (3) missing data and (4) CT participants were already receiving cardiology-led palliative care (possible underestimated benefit).

### What this study adds

This study provides useful feasibility and early safety data; the UCG did not appear to be disadvantaged in terms of survival or 4-month outcomes. Willingness to be randomised was not tested but could be built into a pilot embedded into a phase III trial. Recruitment appeared to be easier from patients known to the research team (UCG). This should be taken into account when researchers are dependent on other clinical teams to identify potential participants although eligible patients may be more inclined to participate in an intervention trial.

A recent US statement^7^ encourages problem-driven palliative care independent of prognosis, citing benefits as improved patient/carer understanding, symptoms/suffering relief, patient-centred decision-making, improved communication, better ACP and bereavement support. Although the study was not designed to assess effectiveness, the data on patient understanding, ACP documentation, fewer nights in hospital and being shifted from secondary to community care show promise – consistent with Component 5 (Box 1).

## Conclusion

A clinical trial investigating the cost-effectiveness of cardiology-led heart failure palliative care is feasible. A future trial should recruit from usual community-based care eligible for but not referred to cardiology palliative care, and identify those needing more intensive palliative care.

## References

[1] Barnes S Gott M Payne S et al Prevalence of symptoms in a community-based sample of heart failure patients. J Pain Symptom Manage 2006; 32(3): 208–216. 16939845 10.1016/j.jpainsymman.2006.04.005

[2] Murray SA Boyd K Kendall M et al Dying of lung cancer or cardiac failure: prospective qualitative interview study of patients and their carers in the community. BMJ 2002; 325(7370): 929. 12399341 10.1136/bmj.325.7370.929 PMC130056

[3] Murray SA Kendall M Boyd K et al Exploring the spiritual needs of people dying of lung cancer or heart failure: a prospective qualitative interview study of patients and their carers. Palliat Med 2004; 18(1): 39–45. 14982206 10.1191/0269216304pm837oa

[4] Pantilat SZ O’Riordan DL Dibble SL et al Longitudinal assessment of symptom severity among hospitalized elders diagnosed with cancer, heart failure, and chronic obstructive pulmonary disease. J Hosp Med 2012; 7(7): 567–572. 22371388 10.1002/jhm.1925

[5] Rogers A Addington-Hall JM McCoy AS et al A qualitative study of chronic heart failure patients’ understanding of their symptoms and drug therapy. Eur J Heart Fail 2002; 4(3): 283–287. 12034153 10.1016/s1388-9842(01)00213-6

[6] Rogers AE Addington-Hall JM Abery AJ et al Knowledge and communication difficulties for patients with chronic heart failure: qualitative study. BMJ 2000; 321(7261): 605–607. 10977838 10.1136/bmj.321.7261.605 PMC27476

[7] Braun LT Grady KL Kutner JS et al Palliative care and cardiovascular disease and stroke: a policy statement from the American Heart Association/American Stroke Association. Circulation 2016; 134: e198–e225. 27503067 10.1161/CIR.0000000000000438

[8] Howlett JG McKelvie RS Costigan J et al The 2010 Canadian Cardiovascular Society guidelines for the diagnosis and management of heart failure update: heart failure in ethnic minority populations, heart failure and pregnancy, disease management, and quality improvement/assurance programs. Can J Cardiol 2010; 26(4): 185–202. 10.1016/s0828-282x(10)70367-6 PMC2886538 20386768

[9] Jaarsma T Beattie JM Ryder M et al Palliative care in heart failure: a position statement from the palliative care workshop of the Heart Failure Association of the European Society of Cardiology. Eur J Heart Fail 2009; 11(5): 433–443. 19386813 10.1093/eurjhf/hfp041

[10] Whellan DJ Goodlin SJ Dickinson MG et al End-of-life care in patients with heart failure. J Card Fail 2014; 20(2): 121–134. 24556532 10.1016/j.cardfail.2013.12.003

[11] National survey of patient activity data for specialist palliative care services: MDS full report for the year 2013–2014, http://www.endoflifecare-intelligence.org.uk/resources/publications/mdsreport2014

[12] Barclay S Momen N Case-Upton S et al End-of-life care conversations with heart failure patients: a systematic literature review and narrative synthesis. Br J Gen Pract 2011; 61(582): e49–e62. 21401993 10.3399/bjgp11X549018 PMC3020072

[13] Gadoud A Kane E Macleod U et al Palliative care among heart failure patients in primary care: a comparison to cancer patients using English family practice data. PLoS ONE 2014; 9(11): e113188. 25423169 10.1371/journal.pone.0113188 PMC4244094

[14] Harrison N Cavers D Campbell C et al Are UK primary care teams formally identifying patients for palliative care before they die? Br J Gen Pract 2012; 62(598): e344–e352. 22546594 10.3399/bjgp12X641465 PMC3338056

[15] Selman L Harding R Beynon T et al Improving end-of-life care for patients with chronic heart failure: ‘Let’s hope it’ll get better, when I know in my heart of hearts it won’t’. Heart 2007; 93(8): 963–967. 17309905 10.1136/hrt.2006.106518 PMC1994396

[16] Brannstrom M Boman K. Effects of person-centred and integrated chronic heart failure and palliative home care. PREFER: a randomized controlled study. Eur J Heart Fail 2014; 16(10): 1142–1151. 25159126 10.1002/ejhf.151

[17] Sidebottom AC Jorgenson A Richards H et al Inpatient palliative care for patients with acute heart failure: outcomes from a randomized trial. J Palliat Med 2015; 18(2): 134–142. 25479182 10.1089/jpm.2014.0192

[18] Wong FK Ng AY Lee PH et al Effects of a transitional palliative care model on patients with end-stage heart failure: a randomised controlled trial. Heart 2016; 11: 1100–1108. 10.1136/heartjnl-2015-308638 PMC4941184 26969631

[19] Rogers JG Patel CB Mentz RJ et al Palliative care in heart failure: the PAL-HF randomized, controlled clinical trial. J Am Coll Cardiol 2017; 70(3): 331–341. 28705314 10.1016/j.jacc.2017.05.030 PMC5664956

[20] Sahlen KG Boman K Brannstrom M. A cost-effectiveness study of person-centered integrated heart failure and palliative home care: based on a randomized controlled trial. Palliat Med 2016; 30(3): 296–302. 26603186 10.1177/0269216315618544

[21] Wong FKY So C Ng AYM et al Cost-effectiveness of a transitional home-based palliative care program for patients with end-stage heart failure. Palliat Med 2017; 32: 476–484. 28434275 10.1177/0269216317706450

[22] Davidson PM Paull G Introna K et al Integrated, collaborative palliative care in heart failure: the St. George Heart Failure Service experience 1999–2002. J Cardiovasc Nurs 2004; 19(1): 68–75. 14994784 10.1097/00005082-200401000-00011

[23] Quill TE Abernethy AP. Generalist plus specialist palliative care–creating a more sustainable model. N Engl J Med 2013; 368(13): 1173–1175. 23465068 10.1056/NEJMp1215620

[24] Daley A Matthews C Williams A. Heart failure and palliative care services working in partnership: report of a new model of care. Palliat Med 2006; 20(6): 593–601. 17060252 10.1177/0269216306071060

[25] Johnson MJ Nunn A Hawkes T et al Planning for end of life care in people with heart failure: experience of two integrated cardiology-palliative care. Br J Cardiol 2012; 19: 71–75.

[26] Johnson M Parsons S Raw J et al Achieving preferred place of death – is it possible for patients with chronic heart failure? Br J Cardiology 2009; 16: 194–196.

[27] Pattenden JF Mason AR Lewin RJ. Collaborative palliative care for advanced heart failure: outcomes and costs from the ‘Better Together’ pilot study. BMJ Support Palliat Care 2013; 3(1): 69–76. 10.1136/bmjspcare-2012-000251 24644330

[28] Bouamerane M-M Saunderson K Mair F. Caring together manualisation report. London: British Heart Foundation; Marie Curie Cancer Care, 2014.

[29] Research Advisory Group. Impact of programme: quantitative data. London: British Heart Foundation/Marie Curie Cancer Research, 2014.

[30] The Criteria Committee of the New York Heart Association. Nomenclature and criteria for diagnosis of diseases of the heart and great vessels, 6th ed. Boston, MA: Little, Brown and Company, 1994.

[31] Abernethy AP Shelby-James T Fazekas BS et al The Australia-modified Karnofsky Performance Status (AKPS) scale: a revised scale for contemporary palliative care clinical practice [ISRCTN81117481]. BMC Palliat Care 2005; 4: 7. 16283937 10.1186/1472-684X-4-7 PMC1308820

[32] Charlson M Szatrowski TP Peterson J et al Validation of a combined comorbidity index. J Clin Epidemiol 1994; 47(11): 1245–1251. 7722560 10.1016/0895-4356(94)90129-5

[33] Bruera E Kuehn N Miller MJ et al The Edmonton Symptom Assessment System (ESAS): a simple method for the assessment of palliative care patients. J Palliat Care 1991; 7(2): 6–9. 1714502

[34] Green CP Porter CB Bresnahan DR et al Development and evaluation of the Kansas City Cardiomyopathy Questionnaire: a new health status measure for heart failure. J Am Coll Cardiol 2000; 35(5): 1245–1255. 10758967 10.1016/s0735-1097(00)00531-3

[35] Jones P Gosch K Yi L et al The KCCQ-12: a short version of the Kansas City Cardiomyopathy Questionnaire. Circ Cardiovasc Qual Outcomes 2013; 6: A248. 10.1161/CIRCOUTCOMES.115.001958 PMC4885562 26307129

[36] Opasich C Gualco A De FS et al Physical and emotional symptom burden of patients with end-stage heart failure: what to measure, how and why. J Cardiovasc Med 2008; 9(11): 1104–1108. 10.2459/JCM.0b013e32830c1b45 18852581

[37] Kind P. The EuroQoL instrument: an index of health-related quality of life. In: Spilker B (ed.) Quality of life and pharmacoeconomics in clinical trials. Philadelphia, PA: Lippincott-Raven, 1996.

[38] Zigmond AS Snaith RP. The hospital anxiety and depression scale. Acta Psychiatr Scand 1983; 67(6): 361–370. 6880820 10.1111/j.1600-0447.1983.tb09716.x

[39] Higginson IJ Gao W Jackson D et al Short-form Zarit caregiver burden interviews were valid in advanced conditions. J Clin Epidemiol 2010; 63(5): 535–542. 19836205 10.1016/j.jclinepi.2009.06.014

[40] Spertus J Peterson E Conard MW et al Monitoring clinical changes in patients with heart failure: a comparison of methods. Am Heart J 2005; 150(4): 707–715. 16209970 10.1016/j.ahj.2004.12.010

[41] Johnson MJ Bland JM Oxberry SG et al Clinically important differences in the intensity of chronic refractory breathlessness. J Pain Symptom Manage 2013; 46(6): 957–963. 23608121 10.1016/j.jpainsymman.2013.01.011

[42] Hussain JA White IR Langan D et al Missing data in randomized controlled trials testing palliative interventions pose a significant risk of bias and loss of power: a systematic review and meta-analyses. J Clin Epidemiol 2016; 74: 57–65. 26718729 10.1016/j.jclinepi.2015.12.003 PMC4910872

[43] Kane PM Murtagh FE Ryan K et al The gap between policy and practice: a systematic review of patient-centred care interventions in chronic heart failure. Heart Fail Rev 2015; 20(6): 673–687. 26435042 10.1007/s10741-015-9508-5 PMC4608978

